# Microbial succession and its correlation with the dynamics of volatile compounds involved in fermented minced peppers

**DOI:** 10.3389/fnut.2022.1041608

**Published:** 2022-10-20

**Authors:** Ding Ma, Yong Li, Chengcheng Chen, Shichao Fan, Yi Zhou, Fangming Deng, Lingyan Zhao

**Affiliations:** ^1^College of Food Science and Technology, Hunan Agricultural University, Changsha, China; ^2^Department of Human Nutrition, Food and Animal Sciences, University of Hawai’i at Mānoa, Honolulu, HI, United States; ^3^Hunan Guotai Foods Co., Ltd., Yueyang, China; ^4^Junjie Food Technology Co., Ltd., Shaoyang, China; ^5^Lameizi Foodstuff Co., Ltd., Yiyang, China

**Keywords:** fermented minced pepper, natural fermentation, microbial diversity, volatile components, correlation analysis

## Abstract

Fermented minced peppers are a traditional fermented food that has a unique flavor due to various microbial communities involved in fermentation. Understanding the changes in microbial communities and volatile components of fermented minced peppers is particularly important to unveil the formation of unique flavor of fermented peppers. In this study, the microbial communities and volatile compounds in fermented minced pepper was analyzed by high-throughput sequencing and GC-MS, as well as their underlying correlations were also established. Results indicated that 17 genera were identified as dominant microorganisms in the fermentation of minced pepper, accompanied by the detection of 64 volatile compounds. Further hierarchical clustering analysis (HCA) displayed that dynamic change of volatile metabolites were involved in the fermentation process, where alkane volatile components were mainly generated in the early stage (3–5 days), and alcohols volatile components were in the middle stage (7–17 days), while ester volatile components were mainly produced in both the early stage (3–5 days) and last stage (17–20 days). Bidirectional orthogonal partial least squares (O2PLS) analysis revealed that 11 genera were core functional microorganisms of fermented minced pepper. *Cladosporium* and *Hansenpora* were significantly correlated with the formation of 9 and 6 volatiles, respectively. These findings provide new insights into aroma profile variation of fermented minced peppers and underlying mechanism of characteristic aroma formation during fermentation.

## Introduction

Fermented minced pepper, a traditional fermented vegetable in the southern region of China, is widely consumed due to its nutritional and sensory properties (1). Aroma is one of the key criteria to evaluate the quality of fermented minced peppers, together with its appearance and taste. Alcohol, ester, and ketone compounds are regarded as the critical components in fermented peppers, which determine its special favor (2, 3). Numerous factors contribute to the quality of fermented peppers, such as biological origin, substrate conditions, microbial composition, and processing methods. Specifically, the composition of used substrates and fermentative microorganisms are the main factors (4). During the natural fermentation, microorganisms play a critical role in generating special flavor characteristics of fermented peppers *via* complex physiochemical reaction of secondary metabolites (5, 6).

In recent years, large numbers studies have been conducted to dig out the underlying mechanism and correlation between microorganisms and aroma and sensory characteristics of fermented food, such as Fu brick tea, cheese, and fermented bamboo shoots, etc. Li et al. (7) has explored the key aroma compounds and microorganisms, and the relationship between volatiles, sensory descriptors and microorganisms of Fu brick tea during processing, and found *Aspergillus*, *Candida*, *Debaryomyces*, *Penicillium*, *Unclassified_k_Fungi*, *Unclassified_o_Saccharomycetales* genera six fungal genera were identified as core functional microorganisms associated with volatile metabolism (7). Zheng et al. (8) identified eight bacterial genera and seven fungal genera was the core microbiota for flavor production of cheese (8). Guan et al. (9) found that *Lactobacillus*, *Clostridium*, *Enterobacter*, and *Akebia* play a crucial role in the formation of the unique flavor formation of suansun by investigating the dynamics of physicochemical parameters, flavor compounds and microbial communities during the natural production of sour bamboo shoots (9). Nature microbial community determines the formation of volatile components of fermented peppers and the quality of product (10). To standardize fermentation and avoid undesirable substance of fermented peppers, it is urgent to apply pure starter cultures instead of traditional natural fermentation. Therefore, it is vital to elucidate the key microbial community in traditional fermented peppers in China, which determine favor aroma and desirable properties of fermented peppers. So far most of the studies have mainly focused on the composition of volatile compounds in traditional Chinese fermented minced peppers or the differential volatile compounds between raw material and finished products. However, the information of the correlation between microbial diversity and flavor profile of fermented minced pepper was still insufficient.

In this study, fermented minced pepper was studied to (a) investigate the bacterial and fungal community during the fermentation process using a high-throughput sequencing method, (b) monitor the changes in volatile flavor components during fermentation using gas chromatography-mass spectrometry, and (c) assess the correlation between volatile flavors and microbial communities during fermentation using bidirectional orthogonal partial least squares (O2PLS) regression. Those findings were valuable for gaining insight into the mechanisms of aroma formation in fermented minced pepper, and improving the quality of fermented minced peppers with desirable sensory properties.

## Materials and methods

### Preparation of fermented minced pepper

Fresh *Capsicum annuum* L. Var. *Dactylus* M were cleaned, minced, salted with 8% (w/w) salt, placed in 24 sterile pickle jars with the same mass, covered, sealed with water, and fermented in a 20°C incubator. To study the changes in microbial diversity during fermentation, three Mason jars were removed at the same time on days 3, 5, 7, 9, 11, 14, 17, and 20 of fermentation for aseptic sampling. For sampling, 100 g of each product was transferred into tubes. minced pepper samples were labeled as 3, 5, 7, 9, 11, 14, 17, and 20 days, and stored at –80°C. Fresh minced pepper was labeled as 0 days.

### DNA extraction, amplification, and sequencing

Total DNA was extracted from all samples with an E.Z.N.A Soil DNA kit (OMEGA, Bio-Tek, USA), according to the manufacturer’s instructions, and stored at –20°C. We used the universal forward primer 27 F (5′-AGAGTTTGATCCTGGCTCAG-3′) and the reverse primer 533 R (5′-TTACCGCGGCTGCTGGCAC-3′) to amplify the V1–V3 region of bacterial 16S rDNA gene. We used a broadly conserved primer set (ITS1 and ITS4) to amplify the ITS region of fungal ITS rDNA. The 454 Life Sciences primer B sequence was found within the forward primer ITS1 (5′-TCCGTAGGTGAACCTGCGG-3′), while the 454 Life Sciences primer A sequence was found within the reverse primer ITS4 (5′-TCCTCCGCTTATTGATATGC-3′). Each PCR product was tagged using a specific 10-nt barcode. The PCR reactions (20 μL) were performed using 5 μM of reverse and forward primers, 10 ng of template DNA, 2 μL of 2.5 mM dNTPs, and 2 μL of 5 × fast Pfu master mix. Thermal cycling was performed as follows: initial denaturation for 2 min at 95°C, 30 cycles of denaturation for 30 s at 95°C, annealing for 30 s at 55°C, and extension for 30 s at 72°C. The final extension was performed at 72°C for 5 min. The replicated PCR products were mixed in a PCR tube, visualized on a 2.0% agarose gel, and purified with an AxyPrep DNA Gel Extraction Kit (AXYGEN), according to the manufacturer’s instructions. Prior to sequencing the PCR product, its DNA concentration was analyzed with a QuantiFluor-ST (Promega, USA) and its quality was determined with an Agilent 2100 bioanalyzer (Agilent, USA). The resulting amplicons from each reaction mix were then mixed, in equimolar proportions, based on their concentrations. Emulsion PCR was then performed to produce the amplicon libraries, according to the methods used by 454 Life Sciences. A 454/Roche A sequencing primer kit was used on a Roche Genome Sequencer GS FLX Titanium platform at Shanghai Majorbio Bio-Pharm Technology Co., Ltd., (Shanghai, China) to carry out the pyrosequencing of the amplicons.

### Bioinformatics analyses

QIIME (Version 1.17^1^) was used to process the resulting raw DNA sequences, while the standard barcodes and primer sets were not included. We trimmed all sequences with quality scores that were lower than 20, while those sequences with lengths less than 200 bp, or which possess ambiguous or homologous base scores less than 6, were removed. We denoised the pyrosequencing data, identified the chimera, and used UCHIME (Version 4.2.40^2^) to remove them from the datasets. Once the low-quality sequences were removed, the sequences of suitable quality were grouped into operational taxonomic units (OTUs) using USEARCH (Version 6.1^3^) and 0.97 cut-off settings. The Ribosomal Database Project (RDP) classifier and NCBI Taxonomy Browser were used to sort the taxonomic classifications of the resulting sequences.

### Extraction of volatile components from minced peppers

The headspace solid-phase microextraction (HS-SPME) method was employed to extract the volatile compounds from the minced pepper samples. The volatile components were analyzed according to the methods previously described (2). Samples (30 g each) were blended with 30 ml of distilled water, and 2 g of the sample was immediately transferred into a 15 ml SPME vial (Supelco, Bellefonte, PA, USA) followed by addition of 50 μL 2-octanol (10^–6^ mol/L) in methanol as an internal standard. After sample preparation, each vial was placed in a water bath at 70°C for 15 min with agitation to reach an equilibrium state. Subsequently, a fiber coated with 50/30 μm DVB/CAR/PDMS (Supelco, Bellefonte, PA, USA) was injected into the vial for 30 min to absorb volatile compounds.

### Determination of volatile components using GC–MS

GC-MS analysis was carried out using a Shimadzu GC-2010 gas chromatograph connected to a QP2010 mass spectrometry system (Shimadzu Corp., Kyoto, Japan). A DB-Wax fused silica capillary column (30 m long × 0.25 mm internal diameter × 0.25 μm film thickness) was used with helium as the carrier gas at a constant flow rate of 1 ml/min. The heating gradient program was 40°C for 2 min, followed by increasing at 4°C/min to 80°C and remaining for 1 min. Thereafter, the temperature was raised to 240°C at 3.5°C/min and held on this stage for 4 min. Helium (purity 99.999%) carrier gas flow was at a constant pressure of 2 psi. All mass spectra were acquired in the electron impact (EI) mode (70 eV ionization energy, source temperature 225°C). EI mass spectra ranged from 30 to 550 a.m.u. Volatile compounds were identified by comparing the mass spectra of the samples with the data system (NIST 08 and WILEY 05). Quantitative results were calculated from the peak areas of the GC-MS chromatograms.

### Statistical analysis

The samples were analyzed in triplicate to generate results in the physicochemical analyses. Significant differences were determined using a one-way ANOVA in SPSS 20.0 (International Business Machines Corp., USA). The line graph was created using OriginPro 2019 (OriginLab Corp., USA). To study the dynamic succession of microbial communities, hierarchical clustering analysis (HCA), principal component analysis (PCA), and Spearman’s correlation coefficient calculations were performed using OriginPro 2019 (OriginLab, Inc., USA). The heatmaps and stacked histogram of the relative abundance of microbes at the genus level were created using OriginPro 2019 (OriginLab Corp., USA). O2PLS modeling was used to outline the relationship between the microbiota and volatile components assessed in this study. This consisted of a simultaneous projection of both the X and Y matrices on low-dimension hyper planes. The *R*^2^ (close to 1) and *Q*^2^ (> 0.4) are both necessary conditions for producing an optimal model and indicate suitable predictive ability. The O2PLS of the multivariate analysis was performed using SIMCA 14.1 (Umetrics, Sweden), while the visualized network planning of the Pearson correlation coefficient was conducted using Cytoscape 3.8.2.

## Results and discussion

### Dynamics and succession of the microbial community

A total of 104,784 16S rDNA and 10,781 ITS rDNA valid reads were generated from nine fermented minced pepper samples, with a total of 3,844 and 967 OTUs for bacteria and fungi, respectively, at an identity level of 97%. As showed in Supplementary Figures 1, 2, Shannon and Simpson diversity index analysis indicated that bacterial diversity in fermented minced pepper was initially increased with fermentation time, and reached maximum at the 9 days. Subsequently, decreased as fermentation prolonged (Supplementary Figure 1). On the other hand, fungal diversity was most abundant in initial time and gradually decreased from 0 to 7 days as fermentation proceeded (Supplementary Figure 2). Additionally, a rapid increase of Chao1 and ACE index was observed from 7 to 9 days. Subsequently, there were decreased from 14 to 17 days, implying that the abundance of fungal species fluctuated during the fermentation.

**FIGURE 1:**
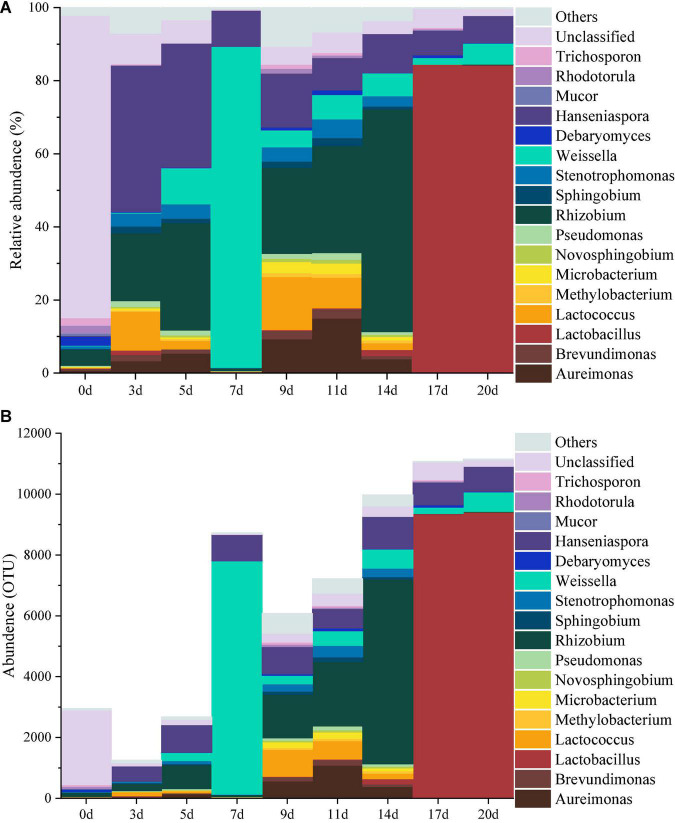
Dynamics of the relative abundance **(A)** and abundance **(B)** of microorganisms at the genus level during the production of fermented minced peppers.

The top 1% of the abundance were used as the main species to study the dynamics of the microbial community during the fermentation of minced peppers. As depicted in Figures 1A,B. Dynamic changes of microbial communities were occurred as fermentation time proceeded. The dominant microorganisms including 12 bacterial and five fungal genera were identified. Initially, the original microbial community in unfermented minced pepper mainly consisted of *Rhizobium* (4%), *Debaryomyces* (2%), *Rhodotorula* (2%), *Trichosporon* (2%), unclassified (83%), and other (2%). During fermentation, the relative abundance of *Rhizobium* was higher at 3–5 days and 9–14 days (13–61%), reached maximum at 14 days. *Hanseniaspora* was the microorganism relatively stable in abundance during minced pepper fermentation. Its growth rate was most rapid during the fermentation from 0 to 3 days, and 40% relative abundance was achieved at 3 days. *Weissella* and *Lactobacillus* were the most abundant microorganisms in relative abundance at 7 days (88%) and 17–20 days (84%), respectively (Figure 1A). Similar results were obtained by the dynamics of microbial genus level abundance during the production of fermented minced peppers (Figure 1B).

**FIGURE 2:**
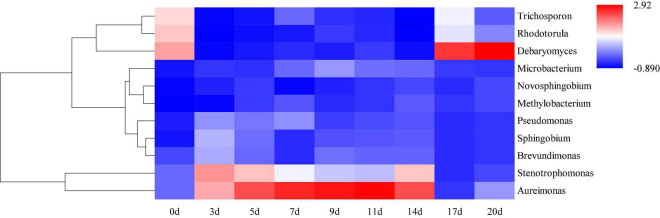
Hierarchical clustering analysis (HCA) of microbial abundance during the production of fermented minced peppers. The colors corresponded to normalized mean levels from low (blue) to high (red).

**FIGURE 3:**
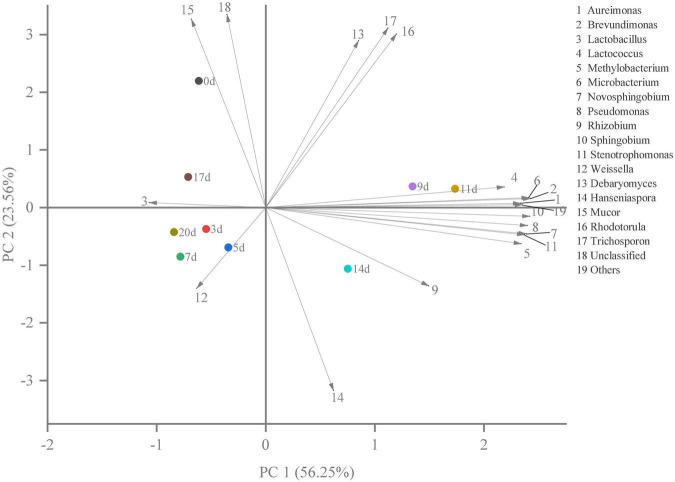
The results of principal component analysis (PCA) showed the correlation between microorganisms and fermentation time during the production of fermented minced peppers.

**FIGURE 4:**
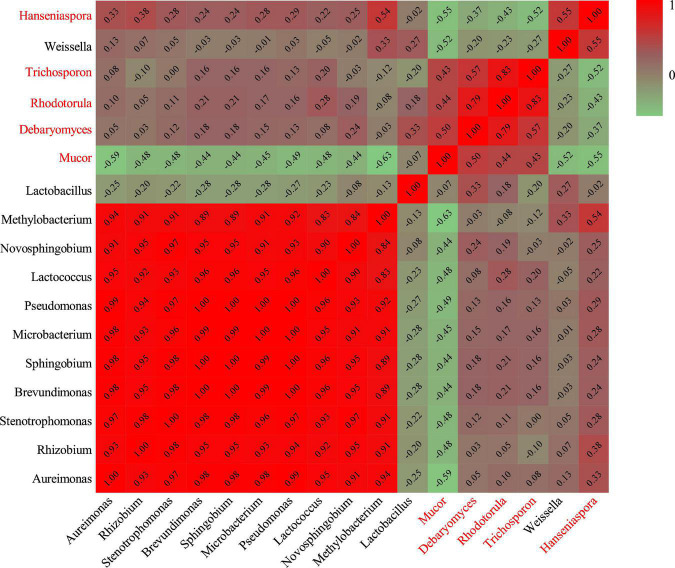
Heatmap of Spearman rank correlation between microorganisms during the production of fermented minced peppers. Black letters are bacteria. Red letters are fungi.

Hierarchical clustering analysis and heat map were used to explore similar growth trends in different microorganisms during fermentation (Figure 2). *Aureimonas* and *Stenotrophomonas*, *Pseudomonas* and *Sphingobium*, *Methylobacterium* and *Novosphingobium*, and *Rhodotorula* and *Trichosporon* displayed similar growth trends during minced pepper fermentation. The variation of microorganisms with fermentation time during minced pepper fermentation was further analyzed by PCA (Figure 3). *Aureimonas* and *Lactococcus* were the main genera in the early fermentation process and were highly correlated with 9 and 11 days. While *Rhizobium* was associated highly with 14 days, *Weisseria* was found to be strongly associated with 5 and 7 days, and *Lactobacillus* was shown to be correlated highly with 17 and 20 days. This result suggests that microorganisms become dominant at different stages of fermentation as they evolve and compete, leading to large variations in microbial abundance and diversity at each fermentation time. Co-occurrence/exclusion analysis is an effective method to elucidate correlations in complex microbial communities (11, 12). The interactions between microorganisms during minced pepper fermentation were shown in Figure 4. Positive correlations were found between bacteria such as *Aureimonas*, *Rhizobium*, *Stenotrophomonas*, *Brevundimonas*, and *Methylobacterium*. While *Lactobacillus* as a bacterium was negatively correlated with most bacteria. For fungal community, positive correlations were found between *Mucor*, *Debaryomyces*, *Rhodotorula*, and *Trichosporon*. *Hanseniaspora*, as a fungus, was negatively correlated with these fungi. In addition, *Mucor* was negatively correlated with all bacteria. No significant correlations were found between *Debaryomyces*, *Rhodotorula*, *Trichosporon*, and *Hanseniaspora* and bacteria.

Indeed, several of the detected genera had previously been detected in dairy products, fermented vegetables, meat, and wine, and may contribute to the formation of nutrients and the unique flavors of fermented foods. *Lactobacillus* plays an important role in the fermentation of many foods, such as dairy products, vegetables, meat, and wine (13). Lactic acid fermentation positively affects the flavor and nutritional content of foods by producing organic acids, bacteriocins and volatile compounds, and contributes to improved sensory and quality safety of foods. *Weissella* is one of the common microorganisms used in the preparation of cheese and fermented vegetables. Several studies have demonstrated the antimicrobial ability of compounds produced by *Weissella* against the growth of Gram-positive and Gram-negative bacteria as inhibitors of phytopathogenic and deteriorating fungal and bacterial growth of fruits and vegetables (14, 15). In addition, some endophytic fungi in peppers play certain roles during fermentation. *Hanseniaspora* are endophytic fungi whose abundance is maintained during fermentation; however, their metabolites may have biological activity. For example, the yeast *Hanseniaspora*, isolated from grapes and grape juice, helps to shorten the fermentation time and reduce the ethanol content of the fermentation product, and increases the total polyphenol and flavonoid content of the wine giving it a higher antioxidant potential (16, 17). *Debaryomyces* is the main yeast used in fermented meat products such as dry fermented sausages. Several studies have demonstrated that *Debaryomyces* contributes to food maturation and aroma presentation, and to the formation of flavor substances such as esters (18, 19). The ability of these detected microorganisms to produce specific flavor compounds has not been systematically investigated. This lack of research has resulted in a lack of development potential for improving existing fermented minced pepper products or developing new products.

### Volatile components change during the pepper fermentation

**FIGURE 5:**
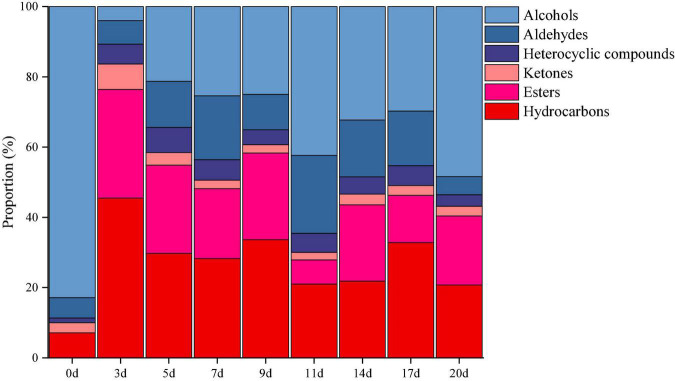
Dynamics of the relative proportion of volatiles during the production of fermented minced peppers.

The volatile components in minced pepper during fermentation were analyzed and quantified by GC-MS. A total of 64 volatile compounds including 17 esters, 14 alcohols, 11 aldehydes, 3 ketones, 16 hydrocarbons, and 3 heterocyclic compounds were identified (Figure 5). Among them, alcohols volatiles were the most abundant, accounted for 4.01–48.43% of the identified volatiles, and followed by hydrocarbons (20.76–45.50%), esters (6.92–30.91%), aldehydes (5.14–22.18%), ketones (2.14–7.25%), and heterocyclic compounds (3.26–7.15%). The alcohol volatile component was highest in unfermented minced pepper (0 days), accounting for 82.87% of the volatile component. As fermentation proceed, the content of alcohols decreased by 34.4%. Previous studies have shown that the significant decrease in alcohol content is partly due to the high volatility of these compounds, leading to their volatilization during fermentation (20). Also, the content of esters and hydrocarbons increased by 19.60 and 13.66%, respectively. This could be caused by microbial metabolism during the fermentation of minced peppers. In addition, the levels of aldehydes, ketones and heterocyclic compounds containing volatiles fluctuated during the manufacturing process, but no significant differences were observed.

**FIGURE 6:**
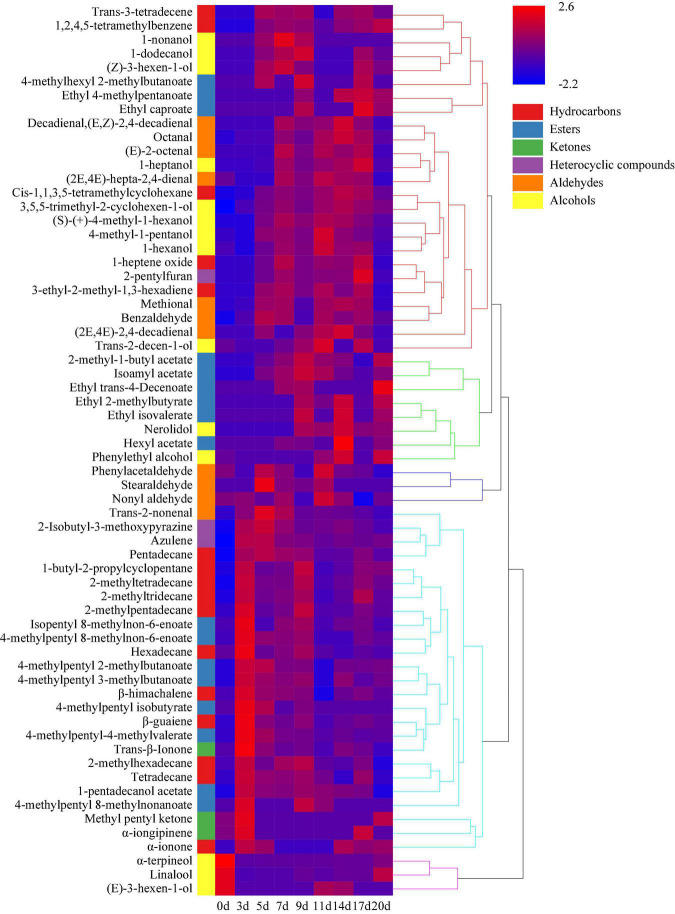
Hierarchical clustering analysis (HCA) of volatile components during the production of fermented minced peppers. The colors corresponded to normalized mean levels from low (blue) to high (red).

Hierarchical clustering analysis classified the volatile components of minced pepper fermentation process into five categories according to their contents at different fermentation times. And heat map analysis showed the changes of different volatile components during minced pepper fermentation (Figure 6). During fermentation, 11 alkanes, 7 esters, 3 ketones, and 2 heterocyclic compounds were found to be higher in 5 days, 8 alcohols, 6 hydrocarbons, 5 aldehydes, and 3 esters in 5–17 days, and 8 esters and 2 alcohols in 17–20 days. These results implied that volatile components of alkanes were mainly produced in the early stage (3–5 days) of fermentation process. Alcohols were mainly generated in the middle stage (5–17 days), while the esters were mainly formed in the early stage (3–5 days) and the late stage (17–20 days). It is noteworthy that some esters were produced late in the minced pepper fermentation processes, which may be due to the fact that esters are synthesized by enzymatic esterification reactions of microorganisms with alcohols and acids as substrates (21). Esters have a sweet or fruity taste and can enhance the flavor of fermented foods by reducing the intensity of unpleasant odors (22). It was found that the concentration of most esters increased significantly after fermentation. Among them, 4-methylpentyl 2-methylbutanoate and 4-methylpentyl 3-methylbutanoate were found to be the most abundant esters in fermented minced pepper. They have an euryhaline herbal odor and a faint waxy odor, respectively (23). Alcohol volatiles have higher gas thresholds than ester volatiles and usually produce fruity and irritating odors, as well as being important precursors to esters (24). Among all alcohols detected in fermented minced pepper, linalool was the most abundant, which has a transient floral and herbal aroma, was detected in large amounts in both unfermented minced peppers, with a gradual increase in content during fermentation. Those results were in accordance with the studies conducted by (2, 25). Linalool which is a key odorant in fermented peppers was derived from glycosides by the action of microbial glycosidases during the fermentation process (26, 27). In addition, β-guaiene was found to be the most abundant terpene volatile component, and this compound contributes significantly to woody flavor and is thought to enhance flavor quality (28). Previous findings have indicated that the content of certain volatiles changes considerably during the fermentation of peppers inoculated with autotrophic or xenobiotic microorganisms (29). Therefore, these changes in volatiles may be related to the metabolism of microorganisms during the fermentation process.

### Correlation between microbiota and volatile components

**FIGURE 7:**
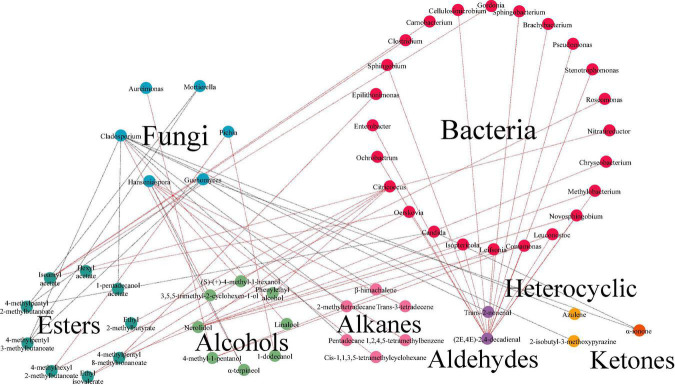
Analyses of correlation between microbiota and volatile components by bidirectional orthogonal partial least squares (O2PLS) modeling during the production of fermented minced peppers. Correlation network between microorganisms and volatile components during fermentation. Red lines indicate positive correlations. Gray lines indicate negative correlations.

Further studies to unveil the underlying correlation between microorganisms and volatiles during the fermentation was conducted. Results of O2PLS model displayed that in fermented minced pepper, there were 45 independent variables X, including 41 genera of bacteria and 4 genera of fungi. And 64 dependent variables Y, with *R*^2^ (0.939) and *Q*^2^ (0.405). The correlation index between microorganisms (X) and volatile compounds (Y) was investigate by O2PLS analysis with Pearson’s correlation method (Supplementary Table 1). And it was visualized in Figure 7. There were 30 genera of microorganisms (24 bacteria and 6 fungi) and 28 volatile components in network. *Cladosporium* was correlated with 9 volatile components (| ρ| > 0.7). But it mere positively related with alcohols. The highest correlation (ρ = 0.902) was with linalool. Similarly, *Hanseniaspora* was correlated with six volatile components (| ρ| > 0.7), including 3 hydrocarbons and 3 alcohols, and only negatively correlated with α-terpineol (ρ = -0.80846). *Citricoccus* was correlated with 4 volatile components (| ρ| > 0.7), including 2 alcohols and 2 esters, and all were positively correlated. In addition, the results showed that a few microbial genera were negatively correlated with volatile components. For example, *Cladosporium* and *Mortierella* showed negative correlations with the differences of seven and two volatile components, respectively. This phenomenon may be due to the decrease in competitiveness between these microorganisms during the fermentation process (30). Furthermore, the selection of core functional microorganisms correlated with flavor from species-rich communities is challenging and requires consideration of both dominance and functionality. To identify the core functional microorganisms in the fermentation process of minced peppers, three criteria were considered: (a) VIP value ≥ 1; (b) correlation coefficient ≥ 0.7; (c) number of microbes highly correlated (| ρ| ≥ 0.7) with chemical compounds ≥ 1 (10) (Supplementary Table 1). Based on these criteria, 11 genera were identified in fermented minced pepper, including *Candida*, *Citricoccus*, *Cladosporium*, *Epilithonimonas*, *Guehomyces*, *Hanseniaspora*, *Mortierella*, *Nitratireductor, Ochrobactrum*, *Oerskovia*, and *Pichia*.

Correlation analysis predicted the relationship between volatile components and microorganisms. In fact, several studies have demonstrated the correlation between microbiota and flavor. *Hanseniaspora* has an important role as a non-Saccharomyces yeasts in the production of aromatic compounds such as esters, higher alcohols, acids, and monoterpenes (31, 32). It has been shown that *H. guilliermondii* produces β-phenylethyl acetate and ethyl acetate (16, 33). *Pichia* can produce flavor substances such as phenylethanol, 2-methylbutyric acid, 3-methylbutyric acid, and ethyl linoleate (34). A significant inverse relationship between acetyl ester hydrolase activity and acetate production was found in *Pichia kudriavzevii* 129 (35). *Candida* was considered important in fermentation because of its ester production capacity, where *Candida antarctica* lipase B (Calb) was found to catalyze the synthesis of several spice esters, including ethyl acetate, isoamyl acetate, *cis-*3-hexenyl acetate, geranyl acetate, ethyl butyrate, isoamyl butyrate, and *cis-*3-hexenyl butyrate (36–38). *Cladosporium* was reported to bioconvert limonene to α-pinoresinol (39, 40). Thus, these core microorganisms may come together to form a core microbiota that contributes to the production of certain key metabolites during the production of fermented minced pepper. Further studies should focus on the mechanisms of volatile component production by functional microorganisms. Meanwhile, the specific expression of related genes during fermentation requires subsequent meta-analysis to monitor how the metabolism of microorganisms affects the formation of key odorants.

## Conclusion

In this study, changes in the main volatile components and the dynamics of the microbial community and the relationships between them were elucidated. The succession and competition of microorganisms during the fermentation process resulted in large differences in microbial abundance and diversity at different fermentation times. A total of 11 microbial genera, including *Candida*, *Citricoccus*, *Cladosporium*, *Epilithonimonas*, *Guehomyces*, *Hanseniaspora*, *Mortierella*, *Nitratireductor, Ochrobactrum*, *Oerskovia*, and *Pichia* were identified as core functional microorganisms. They promoted the production of 14 volatile components such as nerolidol, ethyl isovalerate, 4-methylhexyl 2-methylbutanoate and linalool, which are responsible for providing important fruit or floral aromas to fermented minced peppers. These findings have contributed to the elucidation of the potential role of specific bacterial genera in the formation of specific flavors during fermented minced pepper production, and have helped in the development of fermented minced pepper starter cultures with unique flavors and consistent quality.

## Data availability statement

The original contributions presented in this study are included in the article/Supplementary material, further inquiries can be directed to the corresponding authors.

## Author contributions

FD and LZ designed the work. DM performed the experimental work and prepared the initial draft of manuscript. YL analyzed the data. CC, SF, and YZ critically revised the manuscript. All authors contributed to the article and approved the submitted version.

## Footnotes

1
http://qiime.org/

2
https://www.drive5.com/usearch/manual/uchime_algo.html

3
http://drive5.com/usearch/
