# Supplementary material for: Microbial succession and its correlation with the dynamics of volatile compounds involved in fermented minced peppers
Source: Front Nutr. 2022 Oct 20;9:1041608. doi: 10.3389/fnut.2022.1041608 (PMC9630939; doi:10.3389/fnut.2022.1041608)
Supplement: Supplementary file 2 [file Data_Sheet_1.docx]

**Supplementary figure legends:**

**Supplementary Figure 1**. α-diversity of the bacterial community during the production of fermented minced peppers.

**Supplementary Figure 2**. α-diversity of the fungal community during the production of fermented minced peppers.

**Supplementary Figure 1**

**Supplementary Figure 2**
